# Surgery is associated with better long-term outcomes than pharmacological treatment for obesity: a systematic review and meta-analysis

**DOI:** 10.1038/s41598-024-57724-5

**Published:** 2024-04-25

**Authors:** Leonardo Zumerkorn Pipek, Walter Augusto Fabio Moraes, Rodrigo Massato Nobetani, Vitor Santos Cortez, Alberto Santos Condi, João Victor Taba, Rafaela Farias Vidigal Nascimento, Milena Oliveira Suzuki, Fernanda Sayuri do Nascimento, Vitoria Carneiro de Mattos, Leandro Ryuchi Iuamoto, Wu Tu Hsing, Luiz Augusto Carneiro-D’Albuquerque, Alberto Meyer, Wellington Andraus

**Affiliations:** 1 Department of Neurology, Hospital das Clínicas HCFMUSP, Faculdade de Medicina, Universidade de Sao Paulo, São Paulo, SP Brazil; 2 Faculty of Medicine FMUSP, University of São Paulo, São Paulo, Brazil; 3 Centro Universitário FMABC, Santo André, São Paulo Brazil; 4 Center of Acupuncture, Department of Orthopaedics and Traumatology, University of São Paulo, São Paulo, Brazil; 5 Department of Gastroenterology, Hospital das Clínicas, HCFMUSP, Avenida Doutor Arnaldo, 455, São Paulo, Brazil

**Keywords:** Obesity, Surgery, Long term outcome, Pharmacological treatment, Endocrine system and metabolic diseases, Gastrointestinal diseases

## Abstract

Obesity is a highly prevalent disease with numerous complications. Both intensive medical treatment with the use of pharmacological drugs and bariatric surgery are current options. The objective of this meta-analysis was to compare, in the long-term, intensive medical treatment and surgery based on twelve parameters related to weight loss, cardiovascular and endocrine changes. A review of the literature was conducted in accordance with the PRISMA guidelines (PROSPERO: CRD42021265637). The literature screening was done from inception to October 2023 through PubMed, EMBASE and Web of Science databases. We included randomized clinical trials that had separate groups for medical treatment and bariatric surgery as an intervention for obesity. The risk of bias was assessed through RoB2. A meta-analysis was performed with measures of heterogeneity and publication bias. Subgroup analysis for each surgery type was performed. Data is presented as forest-plots. Reviewers independently identified 6719 articles and 6 papers with a total 427 patients were included. All studies were randomized controlled trials, three had a follow up of 5 years and two had a follow up of 10 years. Both groups demonstrated statistical significance for most parameters studied. Surgery was superior for weight loss (− 22.05 kg [− 28.86; − 15.23), total cholesterol (− 0.88 [− 1.59; − 0.17]), triglycerides (− 0.70 [− 0.82; − 0.59]), HDL (0.12 [0.02; 0.23]), systolic pressure (− 4.49 [− 7.65; − 1.33]), diastolic pressure (− 2.28 [− 4.25; − 0.31]), Hb glycated (− 0.97 [− 1.31; − 0.62]), HOMA IR (− 2.94; [− 3.52; − 2.35]) and cardiovascular risk (− 0.08; [− 0.10; − 0.05]). Patient in the surgical treatment group had better long term outcomes when compared to the non-surgical group for most clinical parameters.

## Introduction

Obesity has been a known condition for over 2000 years ^1^ but that has become much more prevalent in recent decades. Despite great efforts to prevent this disease, the prevalence in adults in the United States has increased in recent decades and reached 42.4% in 2018. The GBD Obesity Study^2^ Collaborators 2015 showed that this increasing trend occurred in more than 70 countries and is highly expressive in adolescents.

The classification of obesity is defined by a body mass index (BMI) greater than 30 kg/m^2^. The psychological damage that many of these patients suffer in a society governed by aesthetic standards is just one of the most visible and immediate consequences of obesity. Mortality from cardiovascular causes and its relationship with BMI has already been widely studied^3^, showing that the risk increases progressively with the increase of the index. Similarly, obesity was associated with a higher incidence of cancer^4^, respiratory^5^ and metabolic^6^ diseases.

In this context, the importance of effective treatment of this condition is clear, reducing mortality and improving the quality of life of these patients. While some benefits are evident with a loss of just 5%^6^ of their weight, many patients require a more expressive loss to reduce the risks associated with obesity.

There are several treatments available for weight loss. Lifestyle changes, low calorie diet and increasing physical activity are the mainstay treatment for all patients^7,8^. Specific weight loss diets and exercise programs have also been developed for this purpose, yielding varying results. Finally, pharmacological, and surgical treatment has gained more attention in recent years for selected patients in whom other measures were insufficient.

Several studies have demonstrated the effectiveness of bariatric surgery in the short and medium term for the treatment of obesity. More recent studies have also shown that new drugs developed for weight loss may be a viable option for the treatment of this disease^8,9^. Comparison of these new drugs with surgical treatment is scarce in the literature and aimed only at evaluating changes related to weight loss in a short period of time.

This systematic review evaluated the hypothesis whether surgical treatment is superior than non-surgical treatment for patients with obesity. We evaluated the long-term effect of these treatments on anthropometric measures (weight, waist circumference, BMI) and on obesity related pathologies (triglycerides, LDL, HDL, total cholesterol, cardiovascular risk, systolic and diastolic blood pressure, HOMA and glycated hemoglobin).

## Materials and methods

This systematic review was carried out in accordance with the items of Preferred Reports for Systematic Reviews and Protocol Meta-Analysis (PRISMA-P)^10^ and assessing the methodological quality of systematic reviews (AMSTAR-2) guidelines^11^. This study was registered by the Prospective Register of Systematic Reviews (PROSPERO, 258667) before the research was carried out.

Drafting of the research question was based on the PICO strategy^12^, considering: P (Patients with obesity with indication for bariatric surgery based on BMI); I (Bariatric Surgery); C (Pharmacological treatment); O (Long term morbidity/mortality—at least 5 years of follow up).

### Eligibility criteria

#### Inclusion criteria

*Types of studies:* Randomized clinical trials.

*Types of participants:* Patients eligible for bariatric surgery, according to the American Society for Metabolic and Bariatric Surgery (ASMBS).

*Types of intervention:* Bariatric surgery or medical treatment.

#### Exclusion criteria

Studies were excluded if they: (1) did not have one group for each type of intervention (surgery or pharmacologic treatment); (2) had a heterogeneous population; (3) did not use a standard assessment method for the entire duration of the study, or did not have pre-assessment; (4) were not related to the question in the review; (5) were in a language other than English, Portuguese or Spanish; (6) were incomplete, unpublished or inaccessible to the authors.

### Types of variables/parameters analyzed

Data was collected and arranged in tables, including the authors name, date and country of publication, number of participants included in the final analysis, sex, age, and body mass index.

### Literature revision

The survey was from inception to October 10, 2023, without language restrictions, in the Medline database (via PubMed), EMBASE and Web of Science.

Using the search tool, we selected MeSH terms from the most relevant publications to conduct a new search to obtain articles that could be included in this systematic review. In addition, a manual search of theses, meetings, references, study records and contact with experts in the field was carried out.

#### Search strategy

The same keywords were used in all databases, according to each database input format.

The search strategy was:

Pubmed:

(Bariatric Surgery) AND ((nonsurgical) OR (Orlistat) OR (phentermine) OR (topiramate) OR (lorcaserin) OR (naltrexone) OR (bupropion) OR (liraglutide) OR (conservative) OR (conventional) OR (Anti-Obesity Agents) OR (Intensive medical)) AND (obesity) → 3024.

Embase:

(Bariatric Surgery) AND ((nonsurgical) OR (conservative) OR (Anti-Obesity Agents) OR (Intensive medical)) AND (obesity) → 4732.

Web of Science:

(Bariatric Surgery) AND ((nonsurgical) OR (conservative) OR (Anti-Obesity Agents) OR (Intensive medical)) AND (obesity) → 1772.

#### Data extraction

The data for each study was extracted independently by two authors. Disagreements were resolved by consensus. If no consensus was reached, a third author was consulted. Data extraction was carried out using the Rayyan tool—https://rayyan.qcri.org/^13^.

All studies were analyzed by their titles and abstracts, according to inclusion and exclusion criteria. If the eligibility criteria was met, the full text would be extracted. All studies eligible for qualitative analysis are described in the “Results” section.

Missing data was clarified by contacting the authors directly.

#### Data validation

The risk of bias for intervention-type studies was analyzed using the guidelines of the Cochrane Back Review Group (CBRG)^14^.

### Statistical analysis

As several studies of sufficient quality were available, a meta-analysis was carried out with measures of heterogeneity and publication bias. The data was presented through forest-plots, according to their statistical relevance.

Characteristics of study participants are presented as means, minimum and maximum values for quantitative variables, and as frequencies and percentages for qualitative variables. The prevalence values and 95% confidence intervals was calculated using the Wilson method To assess the global heterogeneity between the studies, Cochran's Q test was calculated, as well as the I2 (percentage of variation). The results of the studies' association measures and their respective 95% confidence intervals are presented in forest-plots.

Statistical analysis were performed using the Stata/MP 14.0 software for Windows.

## Results

### Study selection

**Figure 1:**
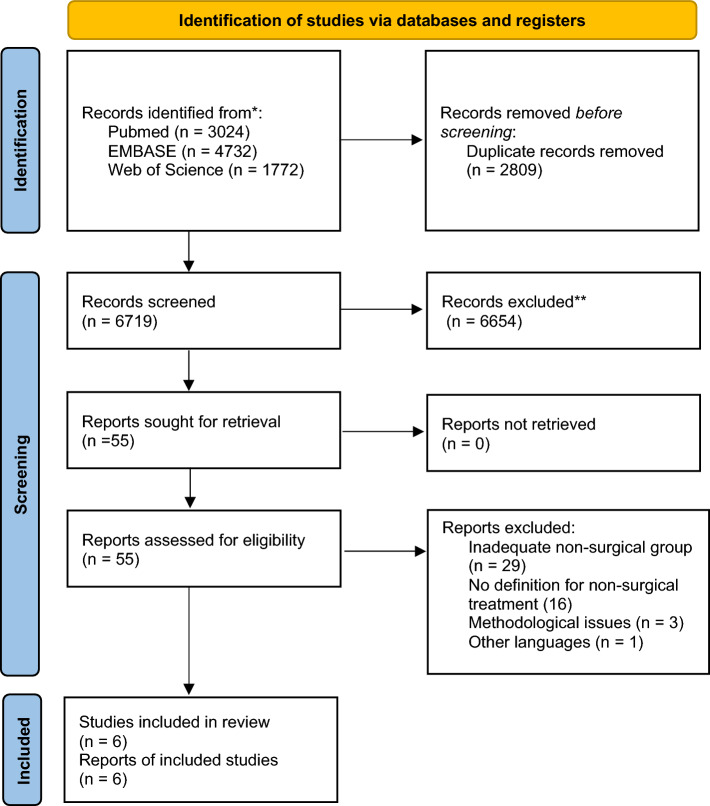
PRISMA 2020 flow diagram for new systematic reviews.

The electronic search found 9528 results for the keywords used. After removing 2809 duplicates and screening through abstract, we considered 55 potentially eligible studies for full-text analysis. Of these, 49 did not respect the exclusion criteria. Only 6 studies were considered eligible for qualitative analysis and 6 articles were eligible for meta-analysis [Fig. 1].

Many studies were excluded due to lack of description for the intervention in the non-surgical group.

### Study characteristics

The following articles were included in the systematic review and meta-analysis^15–20^. In total, there were 427 participants. All studies were RCT. Four had a follow up of five years^15,16,19^ and two had a follow up of 10 years^17,18^. Of the six eligible studies, two were undertaken in the United States of America^15,16^, two in Italy ^17,19^, one in Australia^18^, and one in Singapore^20^. Study characteristics and detailed demographics can be found in Tables 1 and 2. All studies included a group treated exclusively with intensive medical treatment (IMT). The definition of IMT differed between them but were considered if the patients had frequent follow up visits and were instructed on health habits including exercise and diet, with or without the use of pharmacological treatment.

**Table 1:** Study characteristics.

Author, Year	Study type	Period of randomization	Country	Patient initial BMI	follow-up
Cheng 2022	RCT	03/2014–12/2020	Singapore	27–32 kg/m	5 years
Mingrone 2021	RCT	04/2009–10/2011	Italy	≥ 35	10 years
Schauer 2017	RCT	03/2007–01/2011	USA	27–43	5 years
Crawford 2018	RCT	Period not disclosed	USA	27–43	5 years
Mingrone 2015	RCT	04/2009–10/2009	Italy	≥ 35	5 years
O'Brien 2013	RCT	06/2000–11/2000	Australia	30–35	10 years

**Table 2:** Study demographics.

Author, Year		Mingrone 2021	Schauer 2017	Crawford 2018	Mingrone 2015	O'Brien 2013	Chen 2022
Total patients	RYGB	15	49	37	19		12
	BPD	20			19		
	LSG		47	33			
	LAGB					37	
	IMT	20	38	25	15	27	14
Age	RYGB	43.9 ± (7.6)	48.2 ± 8.5	47.4 ± 8.8	not described		40 ± 11
	BPD	43.6 ± (8.2)			not described		
	LSG		48.1 ± 8.1	47.8 ± 7.7			
	LAGB					53.58 ± 6.18	
	IMT	43.5 ± (7.3)	50.2 ± 7.7	51.0 ± 7.6	not described	53.30 ± 8.26	48 ± 9
Sex (% men)	RYGB	40%	42.90%	43.20%	not described		41.7%
	BPD	50%	–		not described		
	LSG		23.40%	24.20%			
	LAGB		–			16.10%	
	IMT	50%	34.20%	32%	not described	40.00%	28.6%
Mean BMI	RYGB	44.2 (41.2–47.8)	37.0 ± 3.4	37.3 ± 3.2	44.0 ± 4.6		29.1 ± 1.6
	BPD	44.4 (39.2–50.6)	–		44.7 ± 7.7		
	LSG		36.0 ± 3.9	35.9 ± 4.1			
	LAGB		–				
	IMT	44.6 (41.6–48.8)	36.4 ± 3.0	36.1 ± 3.1	45.4 ± 6.5		29.7 ± 1.6

There were four modalities of surgery used for weight loss: Roux-en-Y Gastric Bypass (RYGB)^15,17–20^; Biliopancreatic diversion (BPD)^17,19^; Laparoscopic Sleeve Gastrectomy (LSG)^15,16^; Laparoscopic Adjustable Gastric Band (LAGB)^18^. The subgroup analysis for outcomes separated studies in RYGB, LSG and other types of surgery. The non-surgical treatment for obesity included one or the combination of the following medications: Orlistat, Phentermine, Naltrexone, Bupropion, Liraglutide, Lorcaserin, Sibutramine.

### Risk of bias

**Figure 2:**
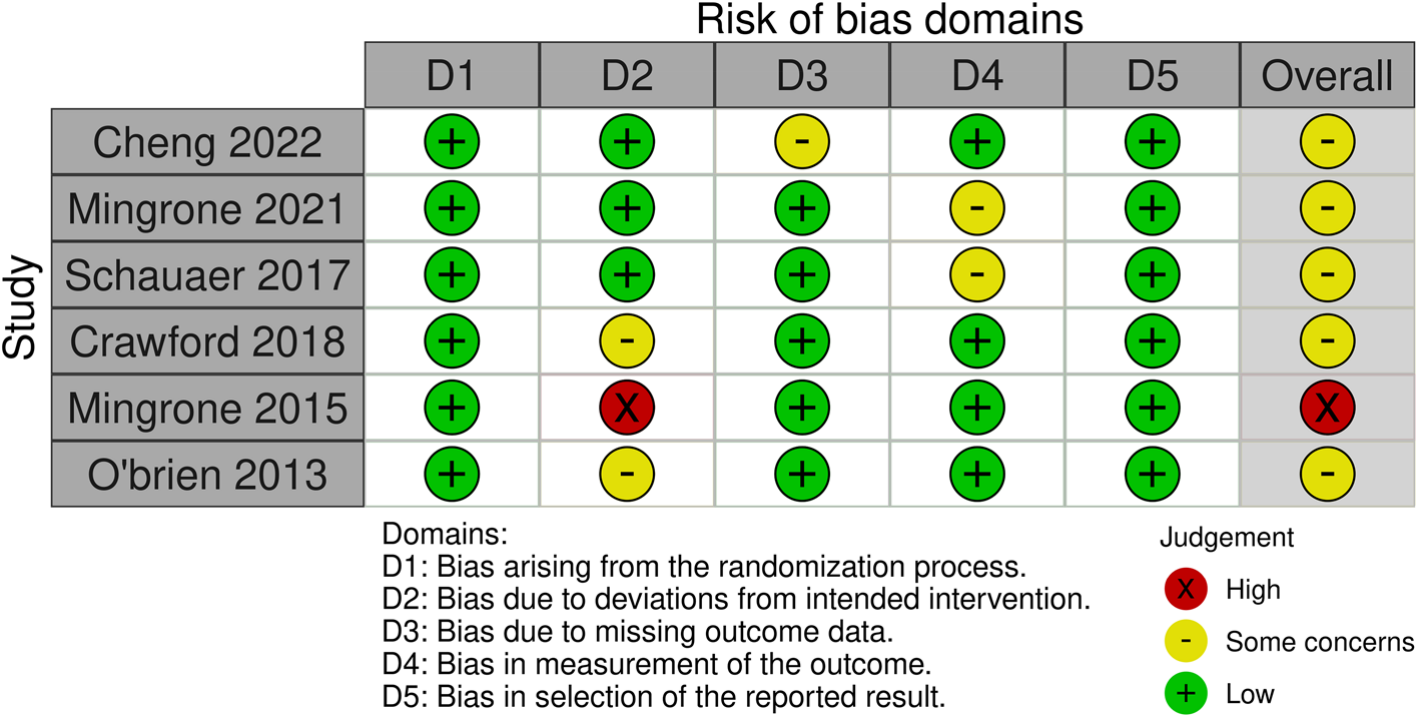
Risk of bias analysis.

After reading the articles included in the systematic review, the following elements were analyzed to determine the level of evidence: study design and selection, detection, loss, reporting and information bias. The summary of the risk of bias analysis for each of the included articles is presented in Fig. 2

All studies had a low risk of bias for most criteria. In three of the studies, assessors were aware of the intervention received by study participants or the information was not available^16,17,20^. Three other studies^15,18,19^ had bias regarding deviations from intended interventions due to the fact that an appropriate analysis to estimate the effects of assignment to intervention was not performed^15^; patients assigned to the control group crossed over to the intervention group, and no measures were reportedly taken to balance that deviation^19^; there was a significant loss of follow-up for all groups^20^.

### Outcomes

#### Weight

**Figure 3:**
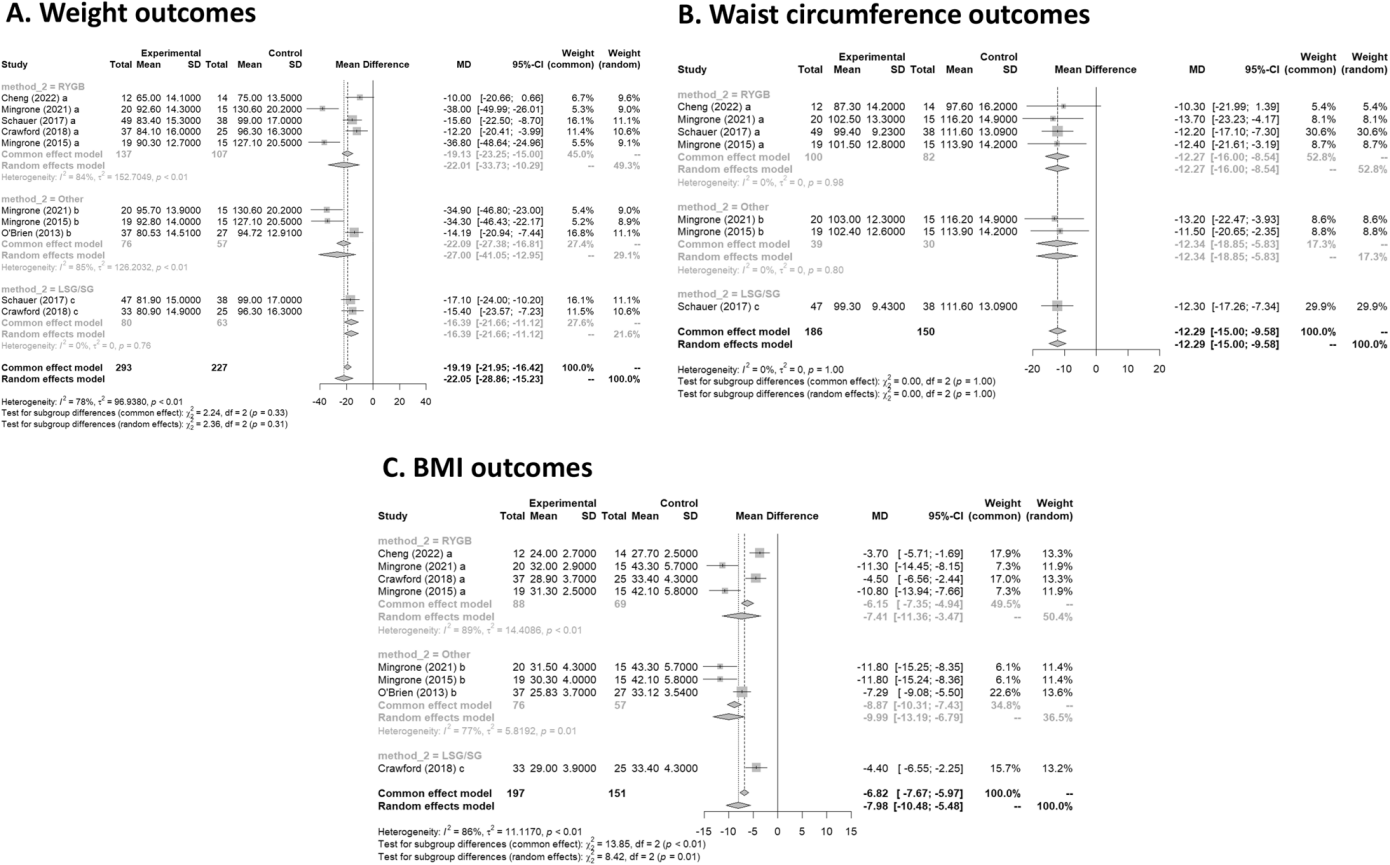
**(A**) Weight outcomes; (**B**) Waist circumference outcomes; (**C**) BMI outcomes.

All six studies had data on weight loss after treatment. Mean difference values and their respective 95% confidence intervals (95% CI) were calculated. In Fig. 3A, the forest plot is shown. All publications found that surgical procedures were more efficient for long term weight loss. The global MD value was − 22.1 kg (95% CI [− 28.9; − 15.2). The measure of heterogeneity I2 (Higgins heterogeneity measure) was 77.8%, a value considered as high heterogeneity. According to Cochran’s Q heterogeneity test, the sample evidence did allow us to reject the null hypothesis of non-heterogeneity (*p* = 0.01).The subgroup analysis showed that there was not a significant difference between the types of surgery (*p* = 0.30).

#### Waist circumference

Four studies had data on waist circumference^16,17,19,20^. In Fig. 3B, the forest plot is shown. Patients treated with surgery had a mean difference of − 12.3 (95% CI [− 15.0; − 9.6]) compared to IMT. The measure of heterogeneity I2 (Higgins heterogeneity measure) was 0%, a value considered as low heterogeneity. According to Cochran’s Q heterogeneity test, the sample evidence did not allow us to reject the null hypothesis of non-heterogeneity (*p* = 0.99).

The subgroup analysis showed that there was not a significant difference between the types of surgery (*p* = 0.99).

#### BMI

Five studies had data on BMI^16–20^. In Fig. 3C, the forest plot is shown. Patients treated with surgery had a mean difference of − 8.0 (95% CI [− 10.5; − 5.5]) compared to IMT. The measure of heterogeneity I2 (Higgins’s heterogeneity measure) was 84%, a value considered high heterogeneity. According to Cochran’s Q heterogeneity test, the sample evidence did allow us to reject the null hypothesis of non-heterogeneity (*p* = 0.01).

The subgroup analysis showed that there was a significant difference between the types of surgery (*p* = 0.01). The group with LAGB and BPD surgery had the highest decrease in BMI, with a mean of − 10.0.

#### Triglycerides

**Figure 4:**
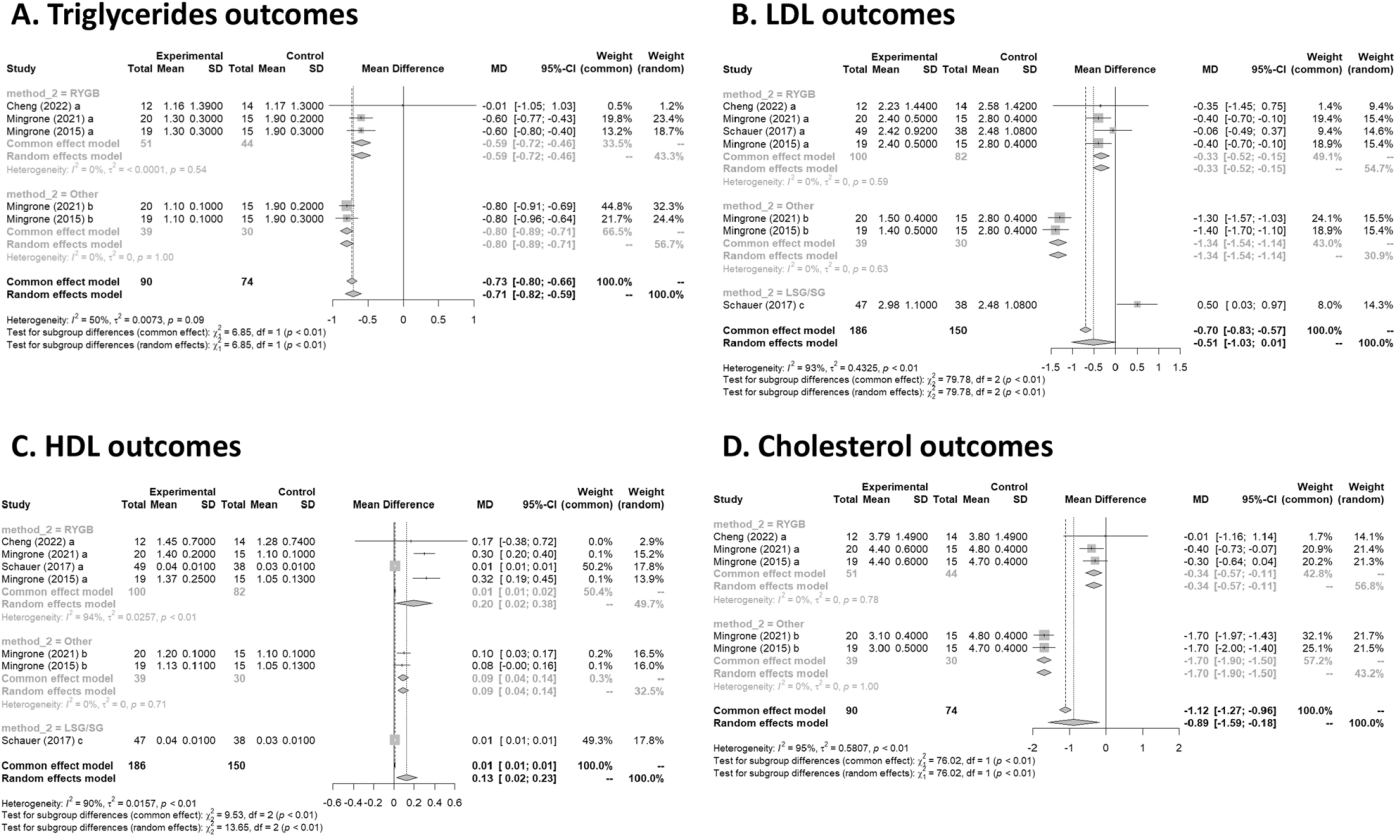
**(A**) Triglycerides outcomes; (**B**) LDL outcomes; (**C**) HDL outcome; (**D**) Cholesterol outcomes.

Three studies had data on tryglycerides^17,19,20^. In Fig. 4A, the forest plot is shown. Patients treated with surgery had a mean difference of − 0.7 (95% CI [− 0.8; − 0.6]) compared to IMT. The measure of heterogeneity I2 (Higgins’s heterogeneity measure) was 50.4%, a value considered high heterogeneity. According to Cochran’s Q heterogeneity test, the sample evidence did not allow us to reject the null hypothesis of non-heterogeneity (*p* = 0.08).

The subgroup analysis showed that there was a significant difference between the types of surgery (*p* = 0.01), with a worse outcome for RYGB.

#### LDL

Four studies had data on LDL^16,17,19,20^. In Fig. 4B, the forest plot is shown. Patients treated with surgery had a mean difference of − 0.5 (95% CI [− 1.0; 0.0]) compared to IMT. The measure of heterogeneity I2 (Higgins’s heterogeneity measure) was 92.7%, a value considered high heterogeneity. According to Cochran’s Q heterogeneity test, the sample evidence did allow us to reject the null hypothesis of non-heterogeneity (*p* = 0.01).

The subgroup analysis showed that there was a significant difference between the types of surgery (*p* = 0.01). There was an increase of 0.5 in LDL for the LSG group. The group with LAGB and BPD surgery had the highest decrease in LDL, with a mean of − 1.3.

#### HDL

Four studies had data on HDL^16,17,19,20^. In Fig. 4C, the forest plot is shown. Patients treated with surgery had a mean difference of 0.1 (95% CI [0.0; 0.2]) compared to IMT. The measure of heterogeneity I2 (Higgins’s heterogeneity measure) was 90.5%, a value considered high heterogeneity. According to Cochran’s Q heterogeneity test, the sample evidence did allow us to reject the null hypothesis of non-heterogeneity (*p* = 0.01).

The subgroup analysis showed that there was a significant difference between the types of surgery (*p* = 0.01). The group with RYGB surgery had the highest significant increase in HDL, with a mean of 0.2.

#### Cholesterol

Three studies had data on cholesterol^17,19,20^. In Fig. 4D, the forest plot is shown. Patients treated with surgery had a mean difference of − 0.9 (95% CI [− 1.6; − 0.2]) compared to IMT. The measure of heterogeneity I2 (Higgins’s heterogeneity measure) was 94.8%, a value considered as high heterogeneity. According to Cochran’s Q heterogeneity test, the sample evidence did allow us to reject the null hypothesis of non-heterogeneity (*p* = 0.01).

The subgroup analysis showed that there was a significant difference between the types of surgery (*p* = 0.01). The group with LAGB and BPD surgery had the highest decrease in cholesterol, with a mean of − 1.7.

#### Cardiovascular risk

**Figure 5:**
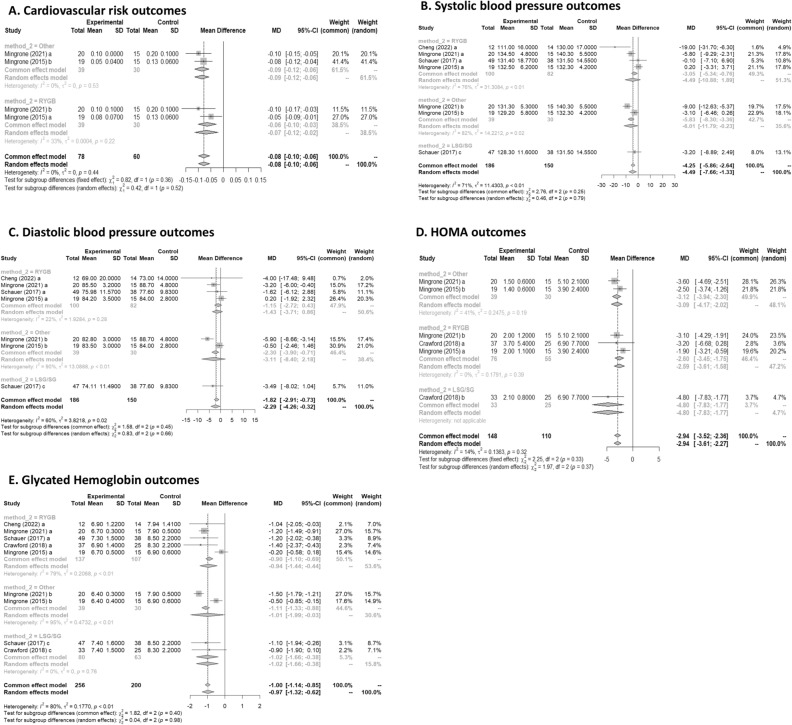
**(A**) Cardiovascular risk outcomes; (**B**) Systolic blood pressure outcomes; (**C**) Diastolic blood pressure outcomes; (**D**) HOMA outcomes; (**E**) Glycated Hemoglobin outcomes.

Two studies had data on cardiovascular risk^17,19^. In Fig. 5A, the forest plot is shown. Patients treated with surgery had a mean difference of − 0.08 (95% CI [− 0.10; − 0.05]) compared to IMT. The measure of heterogeneity I2 (Higgins’s heterogeneity measure) was 0%, a value considered as low heterogeneity. According to Cochran’s Q heterogeneity test, the sample evidence did not allow us to reject the null hypothesis of non-heterogeneity (*p* = 0.44).

The subgroup analysis showed that there was no significant difference between the types of surgery (*p* = 0.36).

#### Systolic blood pressure

Four studies had data on systolic blood pressure^16,17,19,20^. In Fig. 5B, the forest plot is shown. Patients treated with surgery had a mean difference of − 4.49 (95% CI [− 7.65; − 1.33]) compared to IMT. The measure of heterogeneity I2 (Higgins’s heterogeneity measure) was 71%, a value considered as high heterogeneity. According to Cochran’s Q heterogeneity test, the sample evidence did allow us to reject the null hypothesis of non-heterogeneity (*p* = 0.01).

The subgroup analysis showed that there was not a significant difference between the types of surgery (*p* = 0.79).

#### Diastolic blood pressure

Four studies had data on diastolic blood pressure^16,17,19,20^. In Fig. 5C, the forest plot is shown. Patients treated with surgery had a mean difference of − 2.28 (95% CI [− 4.25; − 0.31]) compared to IMT. The measure of heterogeneity I2 (Higgins’s heterogeneity measure) was 60.5%, a value considered as high heterogeneity. According to Cochran’s Q heterogeneity test, the sample evidence did allow us to reject the null hypothesis of non-heterogeneity (*p* = 0.01).

The subgroup analysis showed that there was not a significant difference between the types of surgery (*p* = 0.66).

#### HOMA

Three studies had data on HOMA^15,17,19^. In Fig. 5D, the forest plot is shown. Patients treated with surgery had a mean difference of − 2.94 (95% CI [− 3.52; − 2.35]) compared to IMT. The measure of heterogeneity I2 (Higgins’s heterogeneity measure) was 14%, a value considered as low heterogeneity. According to Cochran’s Q heterogeneity test, the sample evidence did not allow us to reject the null hypothesis of non-heterogeneity (*p* = 0.32).

The subgroup analysis showed that there was no significant difference between the types of surgery (*p* = 0.33).

#### Glycated Hemoglobin

Five studies had data on glycated haemoglobin^15–17,19,20^. In Fig. 5E, the forest plot is shown. Patients treated with surgery had a mean difference of − 1.0(95% CI [− 1.3; − 0.6]) compared to IMT. The measure of heterogeneity I2 (Higgins’s heterogeneity measure) was 79.8%, a value considered as high heterogeneity. According to Cochran’s Q heterogeneity test, the sample evidence did allow us to reject the null hypothesis of non-heterogeneity (*p* = 0.01).

The subgroup analysis showed that there was no significant difference between the types of surgery (*p* = 0.98).

## Discussion

Obesity is defined as a BMI greater than or equal to 30 by the CDC and is currently among the most prevalent diseases in the world, in addition to being an important risk factor for many other diseases. It has high rates of morbidity and mortality^21,22^ and, in this context, weight loss can bring countless positive impacts to the individual. Currently, there are several treatments for obesity, and we can divide them into non-surgical or surgical.

Non-surgical treatments include non-drug and drug treatments. Among the non-medicated, we can highlight the change in eating habits, regular physical exercise, and cognitive behavioral therapy^8^. Ideally, these measures should be implemented for all patients living with obesity, even for those who will undergo drug or surgical treatment. Recently, in addition to lifestyle change, neuromodulation with deep transcranial stimulation has also been studied and has shown effectiveness in weight loss reduction^23^.

A systematic review carried out in 2021, which analyzed 64 articles concluded that among the most effective non-surgical interventions are low-carbohydrate or low-fat diets and combined therapies. This study also showed that non-drug interventions, such as physical exercise, when used alone, are not very effective in reducing the weight of these patients Therefore, a combination of two or more therapies should be chosen^24^.

Pharmacological treatment must be chosen together with the patient. One or more drugs can be used, the main ones used being: Liraglutide, Semaglutide, Tirzepatide, Orlistat, Phentermine and Sibutramine^25^.

Liraglutide was recently approved for the treatment of obesity and is now one of the most widely used drugs. It acts as a GLP-1 receptor agonist^26–28^, enhancing its effects. This group of drugs is already known in the treatment of Type 2 Diabetes Mellitus, a condition that can often be associated with obesity^29,30^, since its pathophysiology involves increased insulin resistance. The main actions of this drug are: increased satiety due to a reduction in the speed of gastric emptying, increased insulin release and decreased glucagon release. Semaglutide is a drug with a similar mechanism of action who demonstrated not only a substantial weight loss^31^, but was also associated with a lower 10-year T2D risk in people with overweight or obesity after 2 years of follow up^32^. More recently, a new drug that combines GLP-1 and GIP receptor agonist, Tirzepatide, has shown even better results in the short term^33^.

Orlistat, in turn, reversibly inhibits the lipase enzyme^34^, which has the function of breaking down fat from food for its absorption, as well as inhibiting the absorption of ingested triglycerides. Thus, there is elimination of fat in the feces^35^. The main adverse effects are gastrointestinal symptoms, however this can be beneficial as it leads to a change in behavior, for example causing a lower consumption of foods rich in fat^36^.

Phentermine, an amphetamine analogue, can be used in conjunction with topiramate for the treatment of obesity. The mechanism of action of the drugs is not yet known, however, significant weight loss has already been observed, in addition to a reduction in the consumption of hypercaloric foods and a decrease in the speed of gastric emptying with the use of this combination of drugs^37,38^.

Sibutramine, widely used in the 1990s, acts to inhibit the reuptake of serotonin, norepinephrine, and dopamine^34^. Serotonin, in turn, activates POMC system neurons and inhibits NPY neurons, thereby promoting reduced appetite and increased satiety. Despite generating weight reduction^39^, some data show increased cardiovascular risk^40^, and therefore, it is no longer used as a first-line drug.

Among the possible surgeries, the most performed today are: Roux-en-Y Gastric Bypass (RYGB), Biliopancreatic diversion (BPD), Laparoscopic Sleeve Gastrectomy (LSG) and Laparoscopic Adjustable Gastric Band (LAGB). According to the NIH and the American Bariatric Society^41,42^, some indications for performing bariatric surgery are adults with BMI greater than or equal to 40 and adults with BMI greater than 35 accompanied by some comorbidity such as type 2 diabetes mellitus, obstructive sleep apnea or hypertension.

RYGB is one of the best-known procedures and its complications vary according to the surgical technique used. Some complications include gastric distention, ulcers, cholelithiasis, hernias, dumping syndrome, and hyperammonaemia encephalopathy.

BPD presents long-term nutritional complications, such as anemia, bone diseases and fat-soluble vitamin deficiency. This technique has high mortality rates, mainly due to the complexity of the technique.

Among the procedures described, LSG is the one with the fewest complications, being described in the literature bleeding or stenosis of the stoma. An alternative technique using endoscopy for sleeve gastroplasty has shown to be safe and efficient for weight loss after 104 weeks, with important improvements in metabolic comorbidities^43^.

The procedure with the lowest mortality rate is the LAGB^44^. Despite this, it can present complications such as obstruction, band erosion, band slippage and gastric prolapse, esophagitis, hernia, in addition to having a high rate of reoperation, reaching 50% of patients who underwent this surgery^45^.

In this article, we compare data on weight loss through intensive drug treatment, which includes changes in eating habits, physical exercise, and medications, and through surgical treatment. Both treatments showed that weight loss caused an improvement in the lipid panel, with a reduction in total cholesterol, triglycerides and LDL, an increase in HDL, improvement in systolic and diastolic blood pressure, decrease in glycated hemoglobin and insulin resistance (accessed through HOMA), in addition to reducing the risk for cardiovascular diseases.

Our systematic review confirmed the findings of individual studies that bariatric surgery has a greater potential for weight reduction, BMI and waist circumference, as already described in individual articles and widely in the literature. It should be noted that even in the long term, this difference remained. Similarly, a 2014 Cochrane systematic review^46^ comparing RCT with more than 1 year of follow-up showed that all 7 articles included demonstrated an advantage of the surgical group. An article^47^ on the use of pharmacological treatment for obesity showed that even recent drugs approved, including GLP 1 agonists, are not able to reduce weight to levels similar to those of bariatric surgery to date, despite the emergence of new drugs still in initial phase^48^. It is worth mentioning that in these studies the comparison time is relatively short (12 months) and that we do not have data on the long-term impact. Thus, in relation to long term weight loss, bariatric surgery is still the best option.

Most articles were not able to individually demonstrate that surgical treatment is superior to non-surgical in terms of pressure reduction. However, the result of the meta-analysis showed a superiority of the surgical group in relation to both systolic and diastolic pressure, more pronounced in the BPD group. Wang^49^ performed a systematic review focused on the impact on pressure and demonstrated that there was a reduction in systolic and diastolic values, but the subgroup analysis showed that this occurs only in the RYGB groups for systolic pressure. Similarly, Schiavon also demonstrated a significant reduction in the need of blood pressure medication after 3 years in the RYGB group when compared intensive medical treatment for obesity^50^. This difference found in only one subtype of surgery seems to be just a reflection of the sample size, which can be interpreted that surgical treatment in general tends to reduce pressure to a greater extent than non-surgical treatment. The fact that different types of surgery are significant may reflect the studies selected in our meta-analysis, which have longer follow-ups.

In relation to both HOMA-IR and glycated Hb, there was a more significant improvement in the group that underwent surgery. The way in which the data on diabetes remission was reported in the articles did not allow a meta-analysis to be carried out with these data and, therefore, it was not included. However, individual data from the Mingrone 2015, Mingrone 2021 and Schauer articles showed that the surgery group had better results. A network meta-analysis from 2021^51^ comparing the different types of metabolic surgery for the treatment of obesity and diabetes showed that RYGB was 20% more likely to result in remission of type 2 diabetes compared to SG. There was no significant difference between the other groups. Moreover, the effects of bariatric surgery on diabetes is not exclusive for patients with obesity, as shown by a study with patients with a BMI of 27–32 kg/m^2^ that had a better glycemic control when treated with RYGB^20^. Regarding the lipid profile, Schauer's study was not able to demonstrate superiority in relation to LDL and HDL parameters. However, by combining the data from Mingrone's articles, it is possible to demonstrate that surgical treatment is superior. Regarding cholesterol reduction, Mingrone's studies showed that although RYGB and BDP were better in relation to non-surgical treatment, the BDP technique had a statistically greater reduction in relation to RYGB. This can be explained by the greater intestinal exclusion in BDP and, therefore, having a greater impact on lipid absorption. Despite Sayeed's study^52^ et al. was not included in this meta-analysis due to the inadequate way of separating the groups for analysis, the results regarding the lipid profile showed that the group that received both interventions was superior to the exclusive non-surgical treatment. It is important to point out that despite a statistically significant difference between the groups, the effect size of this difference is probably not clinically significant.

The choice of treatment for obesity can also have an impact on several other patient comorbidities. Hossain et al.^53^ performed a systematic review with 26 studies that showed that bariatric surgery appears to be more effective in the treatment of asthma. Similarly, a study by Crawford et al.^15^ showed that there is a greater increase in bone turnover in groups undergoing bariatric surgery in relation to pharmacological treatment. Other than that, bariatric surgery is also demonstrated to be superior in the treatment of other obesity related pathologies, such as Non-Alcoholic Steatohepatitis (NASH), and in the treatment of obesity in adolescents^54,55^.

The effect of major cardiovascular adverse events (MACE) and mortality^56^ have also been promising for bariatric surgery. A recent cohort comparing bariatric surgery in patients with obesity and use of GLP1-agonists inpatients with diabetes showed a lower risk of MACE in the surgical group^57^. The surgical treatment has also shown superiority when compared to medical treatment regarding the prevention of diabetic kidney disease in 5 years for patients with diabetes and obesity^58^. Boyers et al. evaluated the cost-effectiveness of surgical and pharmacological treatment in the treatment of obesity and found that RYGB should be the treatment of choice only if the optimization of health system costs is considered^59^.

Another important consideration is the fact that pharmacological and surgical treatment for obesity are not mutually exclusive. Most clinicians choose to combine both treatment modalities in practice to improve results. Weight gain after bariatric surgery is a known possibility, and for those patients, two-thirds of the weight regain can be safely lost with GLP1 agonist, providing clinicians with a therapeutic option for this clinical challenge.

### Methodologies and limitations of the studies

Despite the large number of articles in the literature on the treatment of obesity, there are few RCTs comparing non-surgical and surgical treatment, and most of them only follow up in the short term. In addition, many articles do not adequately describe the strategy used in non-surgical treatment. This lack of data and standardization in this type of treatment can lead to bias and possibly the formation of extremely heterogeneous groups for analysis.

Most of the studies included in our systematic review have diabetes as an inclusion criteria. In this circumstance, our findings may not be generalized to patients with obesity without diabetes.

Another important limitation of our systematic review refers to pharmacological treatment in the non-surgical group. The use of GLP 1 agonists has great potential in the treatment of obesity, but they have only started to be used recently. As the purpose of our article is to assess the long-term impact, there are still few articles available that used this drug. The use of the most recent medications, such as Tirzepatide, could not be evaluated in our study, once there are no RCTs in the literature presenting its long-term effects. Those drugs proved to be very efficient and might have similar effect in the long term. Future systematic reviews may reveal a different results when including the new generation of weight loss medication.

Finally, choosing the most appropriate treatment often involves individual characteristics of each patient, and the impact on quality of life can be extremely subjective and difficult to assess.

## Conclusion

Obesity is a disease that increases the morbidity and mortality of patients, contributing to several secondary diseases. This systematic review evaluated the impact on the main variables related to obesity in the long term. The findings indicated that both treatment modalities are efficacious in managing obesity; however, the surgical group demonstrated superior outcomes in comparison to the non-surgical group across most variables. Nonetheless, the advent of novel pharmacological treatments has shown promising potential. Further studies focusing on the long-term impacts of these new drug treatments should be undertaken to allow for a comprehensive comparison with non-surgical treatment methods.

## Data Availability

Data is provided within the manuscript or supplementary information files.
